# Components of functional diversity revisited: A new classification and its theoretical and practical implications

**DOI:** 10.1002/ece3.10614

**Published:** 2023-10-13

**Authors:** Dénes Schmera, Carlo Ricotta, János Podani

**Affiliations:** ^1^ Balaton Limnological Research Institute Tihany Hungary; ^2^ National Laboratory for Water Science and Water Security Balaton Limnological Research Institute Tihany Hungary; ^3^ Department of Environmental Biology University of Rome ‘La Sapienza’ Rome Italy; ^4^ Department of Plant Systematics, Ecology and Theoretical Biology, Institute of Biology Eötvös University Budapest Hungary; ^5^ Institute of Evolution, Centre for Ecological Research Budapest Hungary

**Keywords:** functional components, functional divergence, functional diversity, functional regularity, functional representation, functional richness

## Abstract

Functional diversity is regarded as a key concept for understanding the link between ecosystem function and biodiversity. The different and ecologically well‐defined aspects of the concept are reflected by the so‐called functional *components*, for example, functional richness and divergence. Many authors proposed that components be distinguished according to the multivariate technique on which they rely, but more recent studies suggest that several multivariate techniques, providing different functional *representations* (such as dendrograms and ordinations) of the community can in fact express the same functional component. Here, we review the relevant literature and find that (1) general ecological acceptance of the field is hampered by ambiguous terminology and (2) our understanding of the role of multivariate techniques in defining components is unclear. To address these issues, we provide new definitions for the three basic functional diversity components namely functional richness, functional divergence and functional regularity. In addition, we present a classification of presence‐/absence‐based approaches suitable for quantifying these components. We focus exclusively on the binary case for its relative simplicity. We find illogical, as well as logical but unused combinations of components and representations; and reveal that components can be quantified almost independently from the functional representation of the community. Finally, theoretical and practical implications of the new classification are discussed.

## 1 INTRODUCTION

There is widespread evidence that functional diversity (FD) has a positive influence on ecosystem productivity, stability, and resilience (Diaz et al., 2007; Tilman et al., 1997). This finding has led to the development of different formulae to measure FD (Mouchet et al., 2010; Petchey & Gaston, 2006). In the simplest cases, a community is envisioned as a unique combination of functional trait states (Podani et al., 2013; Poff et al., 2006; Schmera et al., 2017) or functional types/groups (Diaz & Cabido, 2001), and FD is measured by the number of these functional units. In other approaches, a community is represented by the functional distances among species from which FD is calculated as a total or an average (Heemsbergen et al., 2004; Schmera et al., 2009; Walker et al., 1999). More sophisticated procedures rely on results of multivariate analysis, such as dendrograms (Petchey & Gaston, 2002; Podani & Schmera, 2006), minimum spanning trees (Villéger et al., 2008), or ordinations (Laliberté & Legendre, 2010; Villéger et al., 2008); and quantify FD as a carefully selected property of the given result, i.e. the *functional representation* of the community (e.g. Loiseau et al., 2017; Pavoine & Bonsall, 2011). Advantages and disadvantages of these approaches are emphasized by reviews (Mammola et al., 2021; Mouchet et al., 2010; Petchey et al., 2004; Petchey & Gaston, 2006; Ricotta, 2005).

Mason et al. (2005) pioneered the idea that functional diversity is a multifaceted concept that can be characterized by a limited number of *primary components*. Based on this idea, Villéger et al. (2008) developed an analytical framework for calculating the primary components such as *functional richness* (volume of the functional space occupied by the community), *functional evenness* (the regularity of the abundance distribution in the functional space), and *functional divergence* (deviance of abundance from the center of gravity of the functional space). Thanks to its novelty and soundness, the approach received considerable attention in the ecological literature (e.g. Mouillot et al., 2013). It is important to note that some primary components of Mason et al. (2005) are envisioned with abundance‐weights of species (functional evenness and functional divergence), while others (functional richness) are not.

Pavoine and Bonsall (2011) identified a semantic confusion with the term *functional evenness*. According to the authors, *evenness* is one of the three terms of the *classical species diversity concept* (richness, evenness and diversity, where diversity includes both richness and evenness, see Magurran, 2004; Ricotta, 2007), which defines in general the equitability of the abundance distribution of the diversity units, and not necessarily agrees with the definition of Mason et al. (2005). As a solution, Pavoine and Bonsall (2011) suggested that the term *evenness* should be restricted in general to the equitability of the abundance distribution of diversity units, and the primary component originally termed as *functional evenness* should be called as *functional regularity*. Those authors also argued that functional diversity components incorporating relative abundances should be called *weighted components*, while those disregarding abundance as *unweighted components*.

It follows that the classical diversity concept includes two components (richness and evenness) and three terms (richness, evenness and diversity), while functional diversity involves three components (richness, divergence, regularity), each with abundance‐weighted and unweighted forms (Table 1). Another consequence of this terminology is that the components of functional diversity (i.e. richness, divergence, regularity) should reflect different aspects of functional diversity, no matter whether abundance‐weighted or abundance‐unweighted forms are used. Moreover, the existence of unweighted components suggests that richness, divergence and regularity should conceptualize something different from the evenness component in the classical diversity concept (i.e. the equitability of abundance distribution).

**TABLE 1:** The relationship between components, equitability of abundance distribution and abundance‐weighed components for classical and functional diversity concepts following Pavoine and Bonsall (2011).

Component	Term	Interpretation
*Classical diversity concept*		
Component 1	Species richness	Number of species
Component 2	Species evenness	Equitability of abundance distribution
Two components together	Species diversity	Species diversity (weighted species richness)
*Functional diversity concept*		
Component	Abundance unweighted form	Abundance‐weighted form
Component 1	Unweighted functional richness	Weighted functional richness
Component 2	Unweighted functional divergence	Weighted functional divergence
Component 3	Unweighted functional regularity	Weighted functional regularity

The components of functional diversity are commonly calculated using multivariate techniques, which consider at the outset species as objects and traits as variables. The relationships among species are depicted in numerical and/or graphical form. Pavoine and Bonsall (2011) classified available methods according to whether they rely on *rooted trees*, *minimum spanning trees*, *point configurations in a Euclidean space*, or *distances among species* (see Table 1 in Pavoine & Bonsall, 2011). Surprisingly, this classification has received less attention, notwithstanding that Villéger et al. (2008) also suggested previously the use of *multivariate analyses* (raw functional space and minimum spanning tree) for defining the three primary components of functional diversity. Dealing with phylogenetic diversity, Tucker et al. (2017) discussed three primary components: *richness*, *divergence* and *regularity*. Although the definitions of these components slightly differ from those of Pavoine and Bonsall (2011), the general questions they raised (richness: how much? divergence: how different? regularity: how regular?) are highly relevant to the present subject as well.

Here we provide an overview of the approaches quantifying components of functional diversity. We argue that the components of functional diversity should express different aspects of functional diversity regardless of whether the method is abundance‐weighted or unweighted. As the incorporation of species abundance into diversity indices is challenging due to the wide variety of forms and their mathematical properties (Bulla, 1994; Ricotta et al., 2014; Smith & Wilson, 1996), we focus exclusively on abundance‐unweighted (presence‐absence) methods. Studies of multiple sites (functional beta diversity measures, e.g. Cardoso et al., 2014; Podani et al., 2018; Ricotta et al., 2023) and those incorporating intraspecific trait variability (e.g. Carmona et al., 2019) or occupancy rate (Laini et al., 2023) are also beyond the scope of the present paper.

According to Villéger et al. (2008), functional richness and functional divergence are connected to the raw functional space while functional regularity (originally termed as functional evenness, see Pavoine & Bonsall, 2011) to a minimum spanning tree. Following Mammola et al. (2021), Pavoine and Bonsall (2011), and Tucker et al. (2017), we challenge this strong linkage and argue that the components of functional diversity and the used functional representations of the community are not strictly connected.

In sum, we provide an overview of approaches quantifying functional diversity components, suggest a new classification that generalizes the terms component and representation into a unified framework, examine possible constraints, and discuss the implication of this new classification. The objective of this review is to provide a structured framework showing how the users of functional diversity measures should think about these approaches and, in addition, demonstrating the clear differences between functional representations and functional components. Finally, we argue that the same functional component can be quantified using more or less the same mathematical basis independently from the representation used.

## 2 THE NEW CLASSIFICATION FRAMEWORK

### 2.1 From community data to the functional representations

Let assume that each community is represented by a data matrix of *functional diversity units* (FDUs) by traits. In the evaluation of functionality in ecology, the basic units, the FDUs (defined here as discrete entities representing community members from a functional perspective) are individuals, species, or other taxa with rank preferably not too high above the species level. Features describing the functionality of these units are the traits. Assume that we have *n* traits, each being a well‐defined, measurable property of organisms, usually expressed at individual level and used comparatively across species (McGill et al., 2006). The FDUs‐by‐traits matrices are not always homogenous in measurement scale because a mixture of possible data types (nominal, ordinal, interval and ratio, Anderberg, 1973; Podani, 2000) may appear simultaneously. Moreover, matrices are often incomplete, when no information is available on a given trait for a given FDU or when it is illogical to define a character.

We advise the users (1) to identify the measurement scale of each trait, (2) to record whether the traits contain missing data, and (3) to select an appropriate procedure to obtain the required functional representation. The measurement scale(s) of the variables together with appearance of missing data will define the applicable procedures and consequently the available functional representations. It follows that the quality of the data matrix together with the existing procedures will define which functional representation can be produced. The simplest situation is if each trait is measured on the same scale (the data matrix is homogeneous) and no values are missing. In this case, each functional representation can be produced with the note that the standardization of traits, the selection of the resemblance coefficient, as well as the required methods (e.g. clustering algorithm in case of dendrogram) might have significant influence on the properties of the selected functional representation. If the data matrix is non‐homogeneous in scale, that is, nominal, ordinal, interval, circular and ratio‐scale variables occur simultaneously, and some entries are missing or unknown, the possibilities for data analysis are more limited. For Q‐mode analysis, designed to reveal interrelationships among FDUs as objects, resemblance is readily measured by the Gower (1971) formula and its extensions (de Bello et al., 2021; Pavoine et al., 2009; Podani, 1999; Podani et al., 2023).

### 2.2 Functional representations

**FIGURE 1:**
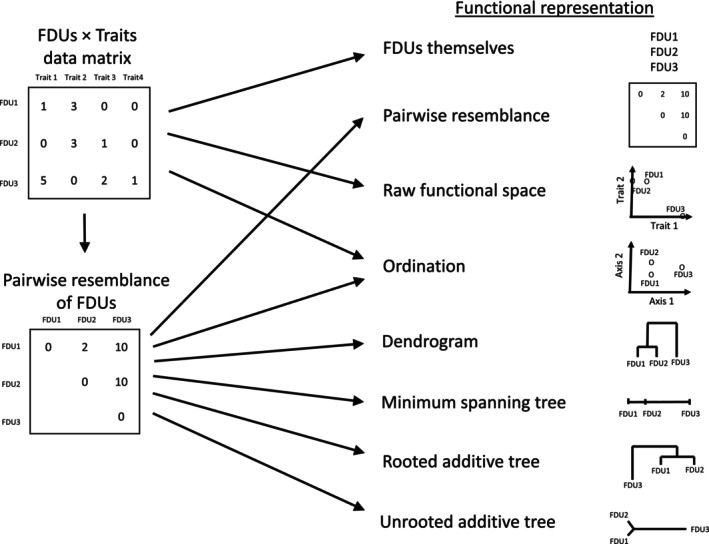
A workflow figure showing a list of functional representations with a simplified roadmap to derive them.

Considering existing measures of functional diversity (see review in Chao et al., 2010; Mammola et al., 2021; Mouchet et al., 2010; Petchey & Gaston, 2006), distinction has been made among seven different functional representations of a community, each corresponding to a well‐defined mathematical object (Figure 1):

1. The list of functional diversity units (FDUs) recognized in the community, mathematically a set abbreviated as U.
2. FDUs as points in the raw functional space with the original functional variables as dimensions, mathematically a rectangular data matrix **X** = {*x*_*ij*_} with FDUs as rows and variables (traits) as columns, a convention followed here.
3. Pairwise dissimilarities (distances) between all possible pairs of FDUs in the raw functional space, mathematically a symmetric matrix, denoted by **D** with its values in set D.
4. FDUs as points in the functional trait space reduced by ordination, represented by a rectangular matrix of coordinates, **C**, with FDUs as rows and dimensions as columns.
5. FDUs as terminal nodes in a rooted tree, a dendrogram, represented by a symmetric matrix of ultrametric distances, **T**.
6. FDUs as nodes of a minimum spanning tree, M: = {V, E} described in terms of a subset V ⊂ U × U of pairs of FDUs and associated distances E ⊂ D.
7. FDUs as terminal nodes of rooted additive trees, described by the symmetric matrix **A** that summarizes pairwise distances along the path connecting each pair of FDUs in the tree. In these trees, the pairwise distances are known to be a better approximation to the original distances than the ultrametric ones. The trees have the property that the terminal nodes are at unequal distances from the root, contrary to dendrograms (Podani, 2000).
8. FDUs as terminal nodes of unrooted additive trees, described by the symmetric matrix **A** that summarizes pairwise distances along the path connecting each pair of FDUs in the tree.

### 2.3 Functional components

We refer to three well‐known components of functional diversity, following Tucker et al. (2017). These are as follows:

1. *Functional richness* (FR) is the size (a measurable quantity) of the functional representation.
2. *Functional divergence* expresses differences among FDUs in the functional representation.
3. F*unctional regularity* reflects the variability of FDUs within the functional representation (“*Variance of*…”, Tucker et al., 2017).

Although the question associated with functional components (Richness: *How much?* / *How many?* Divergence: *How different?* Regularity: *How variable?*) defines the separation of these components, further clarification is needed to assign each method to richness or divergence components. As divergence expresses average differences, it cannot be sensitive to the number of FDUs (whereas a richness measure can). Contrary to this expectation, Pavoine and Bonsall (2011) assign the functional attribute diversity (FAD, Walker et al., 1999) and the modified functional attribute diversity (MFAD, Schmera et al., 2009) to divergence component. Since these are sensitive to the number of FDUs (Mouchet et al., 2010), we assign them to the richness component.

Then, we compile a representation‐by‐component cross‐classification table and assign functional diversity approaches to its cells (Table 2). For some combinations, no procedures can be logically conceived – these are labeled “Not applicable”. In other cases, a technique does exist logically, but it has not yet been used – these are labeled “not yet used”. Below, we first provide some information on FDUs and the functional variables describing them, and then the methods are grouped according to components (columns of Table 2). Note again that we focus on the presence‐absence situation; the description of abundance‐weighted schemes is beyond the scope of this paper.

**TABLE 2:** A summary of multivariate approaches to functional diversity. The first column (rows) show functional representations, columns 2 to 4 functional components, and the cells of the table the different approaches.

Functional representation of the community	Functional richness	Functional divergence	Functional regularity
*How is the community envisioned from a functional perspective?*	*How much?/How many?*	*How different?*	*How variable?*
FDUs themselves	Number of FDUs	Not applicable	Not applicable
	Functional group richness *FGR* (Petchey et al., 2004) Number of functional species (Ricotta, 2005) Number of unique combinations of trait states (Podani et al., 2013; Poff et al., 2006; Schmera et al., 2017) Number of functional units *N* (Schmera et al., 2009)		
Pairwise resemblance between FDUs	Amount of pairwise distances	Average distance, dissimilarity and similarity	Variability in distances
	*Sum of distances among FDUs*, *FAD* (Walker et al., 1999) *Standardized sum of distances among FDUs MFAD* (Schmera et al., 2009) *Sum of shortest trait distances between FDUs* (*nearest trait dispersion*) (Scheiner, 2019)	Mean distance among FDUs *meanD* (Weiher et al., 1998) *Mean shortest trait distance between FDUs* (Ricklefs & Travis, 1980) or *Mean distance to the nearest FDU meanNND* (Weiher et al., 1998)	Variance in the nearest species NNDS2 (Weiher et al., 1998)
FDUs as points in the raw functional space	Volume of the functional trait space occupied	Average distances	Variability of distances
	*Hypercube or hypersphere or hypervolume* (Blonder et al., 2014, 2018; Pla et al., 2012) *Convex hull ConvH* (Cornwell et al., 2006) *FRic* (Villéger et al., 2008)	*Standardized average distance between species and their barycentre: unweighted FDiv* (Villéger et al., 2008)	Mean functional regularity, FRO (Mouillot et al., 2005) Variance of distances between species and their barycentre (not yet used)
FDUs as points in a functional trait space reduced by ordination	Volume of functional trait space occupied	Average distances	Variance of distances
	*Hypercube or hypersphere or hypervolume* (not yet used) *Convex hull* (Céréghino et al., 2018; Diaz et al., 2016)	Average distance between species and their barycentre *Unweighted FDis* (Laliberté & Legendre, 2010)	Overall functional regularity through PCA, OFRO (Mouillot et al., 2005) Variance of distances between species and their barycentre (not yet used)
	Tree length	Mean of branch lengths	Variance of branch lengths
FDUs as terminal nodes in a functional dendrogram	*FD* (Petchey & Gaston, 2002) FDD (Ricotta & Moretti, 2008)	Ricotta and Moretti (2008)	(Not yet used)
FDUs as nodes of a minimum spanning tree	FDM (Ricotta & Moretti, 2008)	Ricotta and Moretti (2008)	Unweighted *FEve* (Villéger et al., 2008)
FDUs as terminal nodes of a rooted additive tree	Sum of path lengths from all terminal nodes to the root (Cardoso et al., 2022)	Mean of path lengths from all terminal nodes to the root (Cardoso et al., 2022)	Variance of path lengths from all terminal nodes to the root (Cardoso et al., 2022)
FDUS as terminal nodes of an unrooted additive tree	Sum of branch lengths (Cardoso et al., 2022)	Mean of branch lengths (Cardoso et al., 2022)	Variance of branch lengths (Cardoso et al., 2022)

#### 2.3.1 Functional richness

Here, the question “How much?/How many” is answered by measuring the size of the functional representation. The number of items in the list of FDUs, i.e., the cardinality of the set U of FDUs is the simplest way for defining FR, abbreviated by *m =* |U|.

Measurement in the raw trait space directly involves the calculation of a volume/range and is most feasible for the interval/ratio scale with no missing values. Even in this case, one must ensure that the measurement units are the same (commensurability); otherwise, some method of standardization/normalization is required. This is implied by standardized PCA, which yields coordinates of FDUs in the ordination space, so that the user may decide to restrict further calculations to meaningful dimensions that explain most of the total variance. If the functional variables are nominal or ordinal then it is hard if not impossible to define a correct formula for calculating volume in the raw space, and the same is true for mixed scale types and data sets with missing values. In practice, therefore, the hypervolume and convex hull approaches require the most detailed type of homogeneous data, which are not always available.

These difficulties are overcome if we switch to the calculation of dissimilarities between FDUs. The sum of values in the semimatrix of dissimilarities is meaningful measure of size and is comparable over different studies if the dissimilarity function ranges between 0 and 1 and the number of FDUs is constant. This is less plausible for dissimilarities and distances that have no upper bound. If the data matrix contains a mixture of different scale types and/or some values are lacking, the use of the Gower formula offers a solution (see Podani et al., 2023, for a procedure applicable to a wide range of variable types). These advantages are “inherited” by multivariate approaches that start from the dissimilarities. Principal coordinates analysis yields ordination scores which are in turn useful in calculating volumes in the ordination space. Hierarchical clustering provides a dendrogram whose total branch length is a widely used measure of size, and the same is true for the minimum spanning trees.

#### 2.3.2 Functional divergence

In most cases, measurement of absolute size is less meaningful especially if different studies are to be compared. Also, the size of any representation provides no information whatsoever on the differences among FDUs. Both problems appear to be solved by the application of divergence measures – which respond to the question of “how different?” These measures operate via calculating averages (means) of all dissimilarities or distances in **D** or E (see Table 2, for all references). For dendrograms, the mean of branch lengths has been suggested (Ricotta & Moretti, 2008). Branch lengths may greatly differ with the clustering method used (Podani & Schmera, 2006), and therefore standardized use of the same algorithm (average linkage) is recommended to ensure comparability of results. For the additive tree, we can calculate two quantities: the mean of branch lengths (unrooted tree), or the mean path length for all terminal nodes from the root (rooted tree). Similarly, the mean may also be obtained for minimum spanning trees. Alternative ways of expressing divergence are to calculate the mean of distances of every FDU from its nearest neighbor in **D**, or the mean of distances from the barycentre of the point cloud based on either **X** (raw space) or **C** (reduced space). Averages do not change linearly over the size of the representation, and therefore they are not suggested for comparisons (Ricotta & Moretti, 2008).

#### 2.3.3 Functional regularity

The same value of any divergence index may result from a situation in which all dissimilarity values are similar and from another case with large discrepancies among the values. It is generally said that in the first case the regularity is low while in the second case high. Functional regularity can thus be expressed directly as the variance of the point cloud based on **X** or **C** (Mouillot et al., 2005) or indirectly as the variance of dissimilarities in **D** (Weiher et al., 1998). Variances may also be calculated for nearest neighbor distances or for distances from the barycentre, similarly to their averages. For dendrograms, there is a measure of variance of distances of all interior nodes to the root. For additive trees, we have two possibilities: the variance of all branch lengths (unrooted tree) and the variance of distances of all terminal points, i.e. FDUs from the root (rooted tree). The first option applies to minimum spanning trees as well.

It is important to note that a further step in the development of the methodology is to combine different components into a single approach. Zhang et al. (2021), for instance, used minimum spanning tree as representation and combined functional richness and functional variability into a singe index (Functional extension and evenness index). In the present paper, we do not discuss such approaches.

### 2.4 The unified framework

**FIGURE 2:**
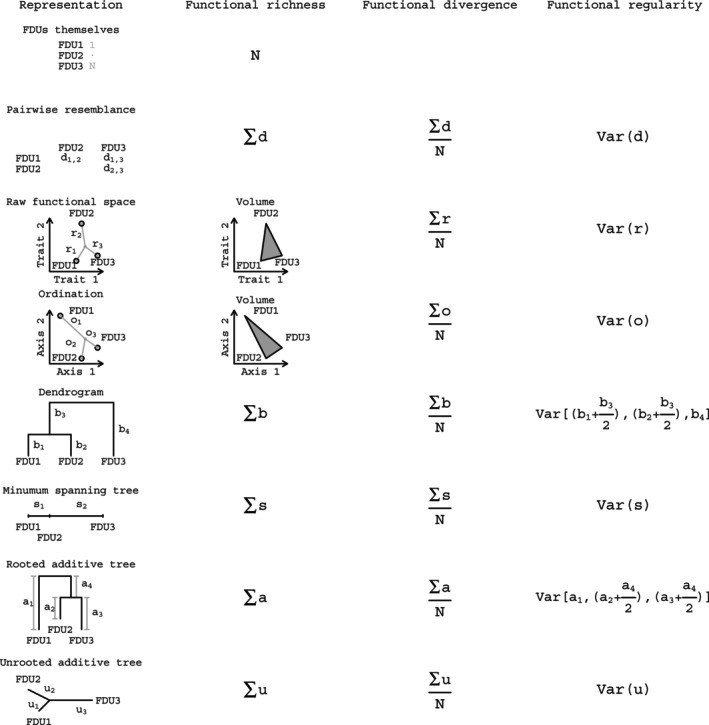
A unified framework where functional richness, divergence and regularity is quantified in a unified way independently from the used functional representation.

We have clarified above the definitions of the three basic functional diversity components (richness, divergence and regularity), and presented a new classification of existing approaches suitable for quantifying these components. Here, we show a unified framework of these approaches, which show that functional richness can be quantified as the “sum”, functional divergence as the “mean”, while functional variability as the “variance” of a property of the given representation (Figure 2). Note that there are different ways to calculate length of tree branches. In the unified framework (Figure 2), we used the fair proportion index (Hartmann, 2013) following the evolutionary study of Jetz et al. (2014).

## 3 DISCUSSION

Mason et al. (2005) pioneered the idea that functional diversity is a multifaceted concept that can be characterized by a limited number of primary components. Based on this idea, Villéger et al. (2008) quantified primary components such as functional richness, functional evenness and functional divergence. The present paper emphasizes that multivariate techniques providing the background of these approaches symbolize the functional representation of the communities, and thus these representations are not strictly connected to the components of functional diversity as assumed until now. In sum, we present a new framework that unifies the representations and components of multivariate functional diversity and the various methods for the quantification.

According to Villéger et al. (2008), functional richness and functional divergence components are connected to the raw trait space, while functional regularity to a minimum spanning tree. Synthetic overviews already realized that functional components have more general meanings (Mammola et al., 2021; Pavoine & Bonsall, 2011; Tucker et al., 2017). The present paper attributes a more general and operative meaning to functional component thereby liberating it from the functional representation of communities.

The new classification framework (Table 2 and Figure 2) showed that some combinations of functional representations and components cannot be logically defined. For instance, if a community is envisioned by the FDUs themselves, then functional divergence and functional regularity cannot be calculated, because the existence of the FDUs does not hold information about differences among them (Petchey & Gaston, 2006). It follows that functional representation and functional component are not fully independent. Our review (Table 2) showed also that there are certain approaches, which are logical but not yet used in functional diversity research. We by no means argue that they have to be used in the future, but their application could contribute to a systematic assessment of functional diversity. To fill this knowledge gap, we suggested new representations and approaches (Table 2 and Figure 2).

The most recent and comprehensive synthesis of functional diversity statistics has been given by Pavoine and Bonsall (2011). They categorized the functional attribute diversity (FAD, Walker et al., 1999) and the modified functional attribute diversity (MFAD, Schmera et al., 2009) as functional divergence, while the present framework categorizes them as functional richness. Based on the unified framework (Figure 2), it is easy to see that FAD is the sum of pairwise distances and thus expresses richness. MFAD is a modified version of FAD that is less sensitive to the number of species but still quantifies the sum of distances.

It is important to note that the representation and component together do not necessarily define unambiguously the measure of functional diversity (Table 2). In many cases, multiple analyses can be applied (Table 2), each with different mathematical properties (Mouchet et al., 2010; Pavoine & Bonsall, 2011; Petchey et al., 2004; Petchey & Gaston, 2006; Ricotta, 2005; Tucker et al., 2017). Fortunately, there are already several studies navigating users among the different approaches (de Bello et al., 2013; Loiseau et al., 2017; Mouchet et al., 2008, 2010; Podani & Schmera, 2006, 2007).

Although the framework of Villéger et al. (2008) has significant contribution to our understanding of the concept of functional diversity, we feel that at least one of its aspects has to be reconsidered. As already mentioned, Villéger et al. (2008) defined three components of functional diversity based on two different functional representations of a community. We argue, however, that the choice of the functional representation of the community may influence the results and conclusions obtained from any approach quantifying functional diversity. We argue that any observed relationship between two components can come from the differences of the components, as well as from the differences of the representations. It follows that if the objective is to characterize several functional components simultaneously, then the use of the same functional representation is required. The framework of the present paper may serve as a guide to ensure comparability of results (Table 2 and Figure 2).

The new framework presents several functional representations (Table 2 and Figure 2), raising the logical question of which one is the best? Unfortunately, the answer is rather complicated and we suggest that the optimum depends on study objectives and the data available. If a community is envisioned as a set of FDUs themselves, then only the functional richness component can be conceptualized. The other representations, however, allow exploring three well‐defined components (richness, divergence and regularity) of functional diversity. Tree representations require special attention. In studying phylogenetic diversity, they are favored understandably (Tucker et al., 2017) while functional diversity is less firmly associated with trees, and further studies are required to identify the strong and weak points of the different representations.

## 4 CONCLUSIONS

We have shown that these approaches dealing with functional components can be classified by considering how the functionality of the community is envisioned, called functional representation, and according to the facet of the functional diversity called functional components. The present paper combines these classifications and presents a unified framework for describing techniques for quantifying functional diversity. Finally, theoretical and practical implications of the new classification are discussed.

## AUTHOR CONTRIBUTIONS

**Dénes Schmera:** Conceptualization (equal); methodology (equal); writing – original draft (equal). **Carlo Ricotta:** Conceptualization (equal); methodology (equal); writing – original draft (equal). **János Podani:** Conceptualization (equal); methodology (equal); writing – original draft (equal).

## Data Availability

There are no data used in this paper.
